# Neurological adverse events associated with baclofen: a pharmacovigilance study based on FDA adverse event reporting system

**DOI:** 10.3389/fphar.2025.1569602

**Published:** 2025-05-14

**Authors:** Yunhan Zhao, Haoxiang Hu, Jiesheng Mao, Jianghai He, Yihan Zhang, Xiaokai Yang

**Affiliations:** Postgraduate Training Base Alliance of Wenzhou Medical University (Wenzhou People’s Hospital), Wenzhou, China

**Keywords:** adverse events, drug safety, FAERS, baclofen, disproportionality analysis, pharmacovigilance, TTO

## Abstract

**Background:**

Baclofen, a centrally acting muscle relaxant, is widely utilized for the management of muscle spasms and alcohol use disorders associated with conditions. However, its neurological safety and tolerability in a large population remain limited. This study aimed to assess the neurological safety and potential risks of baclofen in the real world.

**Methods:**

Data covering the period from the first quarter of 2004 to the third quarter of 2024 were collected from the Food and Drug Administration Adverse Event Reporting System (FAERS). Four disproportionality analysis methods were employed: the Reporting Odds Ratio, the Proportional Reporting Ratio, Bayesian Confidence Propagation Neural Network, and the Multi-item Gamma Poisson Shrinkage (MGPS). These methods were used to detect and evaluate adverse events Adverse drug events associated with baclofen. Additionally, the time to onset analysis was conducted.

**Results:**

A total of 432 neurological-related preferred terms (PTs) were identified. The number of PT that were positive for all four algorithms was 40, and the top 5 PT were Hypotonia, Encephalopathy, Coma, Unresponsive to stimuli, and Cerebrospinal fluid leakage. The top 5 PTs for ROR values are Intracranial hypotension [ROR 66.24 (55.45–79.13)], Cerebrospinal fluid leakage [ROR 51.34 (45.84–57.51)], Autonomic dysreflexia [ROR 47.4 (32.27–69.63)], Basal ganglion degeneration [ROR 33.03 (18.54–58.84)], Sciatic nerve palsy [ROR 21.6 (11.14–41.87)]. The median onset time for baclofen -related ADEs was 27 days. Most cases (n = 241, 55.5%) occurred within the first month of baclofen administration. In an analysis of severe vs. non-severe ADEs, the study found that the incidence of severe cases was higher than that of non-severe cases, with no gender-related differences observed.

**Conclusion:**

This study identified clinically significant PTs using four different algorithms and performed gender subgroup analysis. The TTO analysis indicated that the onset of most ADEs occurred within 27 days. Furthermore, the frequency of severe ADEs was higher than that of non-severe ones. Clinicians should closely monitor for neurological adverse effects caused by baclofen, particularly severe ADEs, and consider individualized dosing strategies. Further research based on real-world data is needed to validate these findings.

## 1 Introduction

Baclofen is a centrally acting muscle relaxant that diminishes neuronal excitability by selectively activating GABA-B receptors within the central nervous system, thereby effectively alleviating muscle spasms and stiffness (Finnimore et al., 1995; Creamer et al., 2018a). Given its substantial clinical efficacy, baclofen has been extensively utilized in managing spasticity associated with conditions such as multiple sclerosis, stroke, and spinal cord injury (Kofler et al., 2009; Creamer et al., 2018a; De Sousa et al., 2022; Shkodina et al., 2024). Furthermore, baclofen serves as an adjunctive therapeutic option for alcohol dependence, effectively alleviating alcohol cravings and reducing the risk of relapse (Garbutt et al., 2021; Agabio et al., 2023). Baclofen can be administered via oral ingestion or intrathecal injection. Oral administration is typically suitable for patients with mild to moderate spasticity, whereas intrathecal injection is indicated for those with severe symptoms or inadequate responses to oral therapy (Bonouvrié et al., 2019).

However, the mechanism of action of baclofen also renders it closely associated with various neurological adverse reactions. Studies have demonstrated that baclofen may induce severe adverse effects, including somnolence, dizziness, confusion, seizures, and even coma, particularly when administered at high dose (Caron et al., 2014; Farhat et al., 2020; Ghannoum et al., 2021). Adverse drug events (ADEs) not only compromise patients’ quality of life but may also result in serious clinical outcomes and impose an additional healthcare burden (Patton and Borshoff, 2018). While the common adverse effects of baclofen have been extensively documented, there remains a paucity of studies addressing rare reactions, events not specified in the drug’s package insert, and their associated risks across diverse patient populations. Therefore, a systematic evaluation of baclofen’s adverse reactions is crucial for optimizing clinical medication strategies and enhancing patient safety.

The FAERS database is one of the largest global platforms for monitoring adverse drug reactions, encompassing millions of patient medication records and associated adverse event reports (Yang et al., 2024). It collects standardized real-world data to support the FDA’s safety monitoring programs for drugs through spontaneous reports submitted by consumers, healthcare professionals, drug manufacturers, and other nonmedical individuals (Ding et al., 2025; Zhu et al., 2025). FAERS offers real-time updates, encompassing an extensive array of drugs and patient populations, while maintaining high levels of data transparency and accessibility (Gong et al., 2024; Li et al., 2025). Its robust signal detection capacity renders it a crucial instrument in pharmacovigilance studies, particularly in the discovery of drug-related adverse events and the evaluation of drug safety. Disproportionality analysis is a powerful tool for pharmacovigilance and drug safety. When disproportionality analysis is applied to the FAERS databases, it helps identify signals for rare adverse events by comparing the observed number of reports for a specific drug-adverse event pair with the expected number (Bate and Evans, 2009).

Given that the adverse reactions of baclofen have not been fully clarified, this study, based on real-world data from the FAERS database, uses disproportionality analysis to identify risk signals in adverse reaction reports related to baclofen, with a focus on analyzing the neurological adverse reactions caused by baclofen. We applied the signal detection method to identify under-reported rare adverse reactions, studied the characteristics of the time to onset (TTO), and analyzed the risk differences between different genders. Our study is intended to offer elaborate information regarding the adverse reactions of baclofen, thereby compensating for the existing research deficiencies and facilitating the provision of valuable guidance for clinicians in the utilization of baclofen and the promotion of patient safety.

## 2 Materials and methods

### 2.1 Study design and data source

The FAERS database is a widely accessible database for postmarketing safety surveillance that collects ADEs from health professionals, drug manufacturers, and patients. As a global database, FAERS covers information on adverse drug events from all over the world, providing an important basis for the safety assessment and regulation of drugs (Li B. et al., 2024). The FAERS database encompasses seven sub-datasets: DEMO (Demographic information), which includes patient age, gender, weight, and other relevant details; DRUG (Drug information), detailing drug names and their roles (primary, secondary, or concomitant drugs); REAC (Adverse event information); OUTC (Patient outcome information), documenting outcomes such as hospitalization and death; RPSR (Report source), identifying the origin of the report (e.g., healthcare professionals or consumers); THER (Treatment dates), specifying the start and end dates of medication; and INDI (Indication for use), indicating the therapeutic purpose of the drug (Wan et al., 2024). ADEs were coded in the FAERS database with the use of the Medical Dictionary for Regulatory Activities (MedDRA), version 27.0 (Zhou et al., 2024). The terminology system of MedDRA is divided into five levels: System-organ Class (SOC), high-level group term (HLGT), high-level term (HLT), preferred term (PT), and lowest level term (LLT). Drugs in the FAERS database are classified into four types: PS (major suspicion), SS (minor suspicion), C (concomitant medication), and I (drug interaction). The primary outcomes of patients included death (DE), life-threatening events (LT), hospitalization (including primary or prolonged hospitalization) (HO), disability (DS), congenital anomalies (CA), and other important medical events (OT). We defined serious outcomes as death, life-threatening, hospitalization, and disability, and the rest as non-serious outcomes.

### 2.2 Data mining and cleaning

**FIGURE 1:**
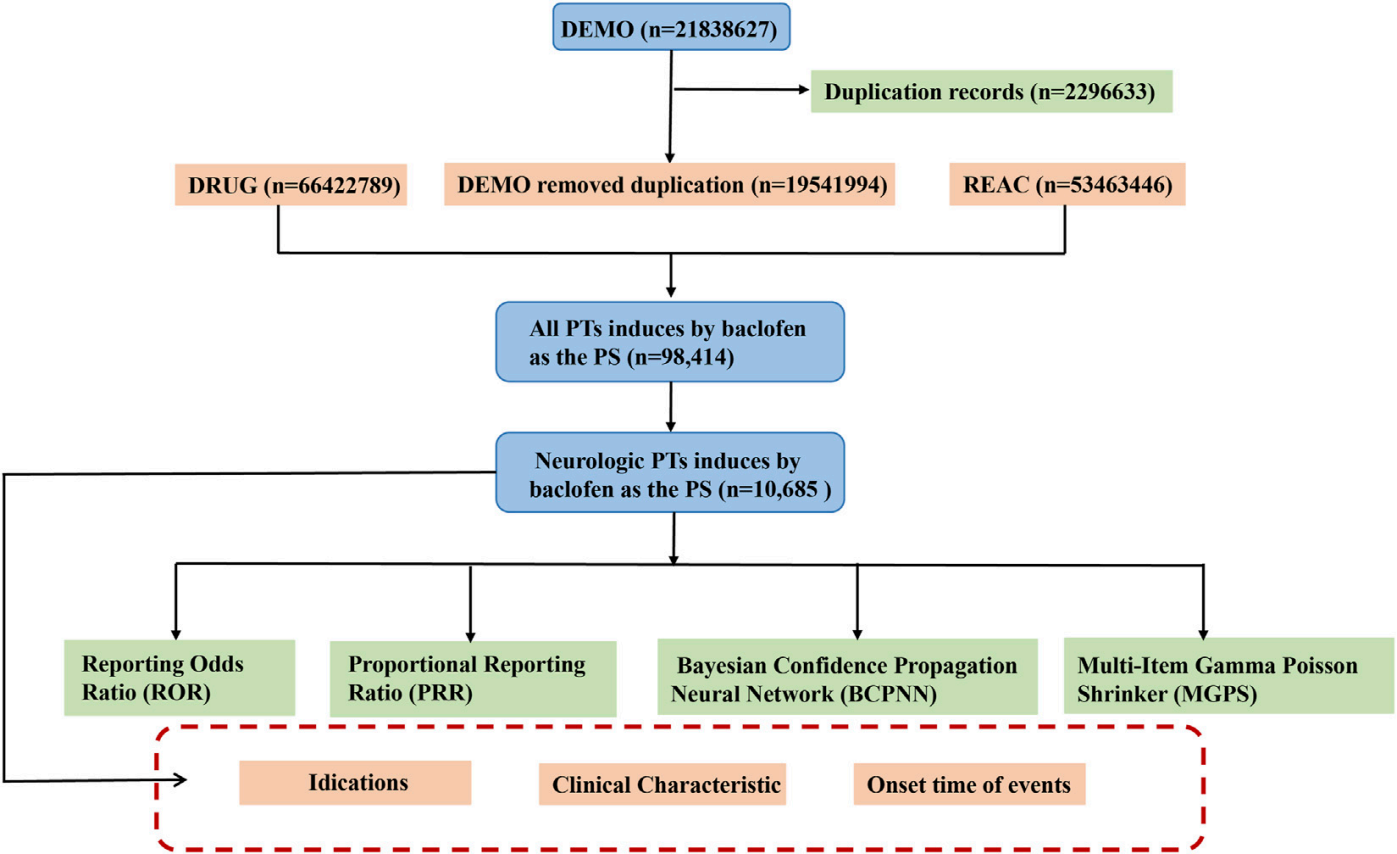
The schematic flowchart of data extraction, processing, and analysis. Footnotes: Abbreviation: DEMO, Demographic information; DRUG, Drug information; REAC, Adverse event information; PT, preferred terms; PS, major suspicion.

During the data mining and cleansing process, we implemented the following systematic steps to enhance data quality and analytical accuracy. Given that FAERS data may contain duplicate reports, we cleaned the data according to FDA-recommended standards (Huang et al., 2024). Specifically, if CASEID was identical, the most recent FDA_DT (report date) was retained. If both CASEID and FDA_DT were identical, the higher PRIMARYID (report identifier) was kept. This process ensured the uniqueness and reliability of the data. In this study, we selected the reports in which baclofen was identified as the PS. Both brand names (Lioresal) and generic names (Baclofen) are utilized to identify records associated with baclofen. All adverse effect terms were standardized using MedDRA version 27.0, and analyses were stratified accordingly. These cleansing procedures not only enhance the reliability of the data but also safeguard the integrity of subsequent signal mining outcomes. Ultimately, we acquired a refined and high-quality baclofen ADEs dataset suitable for in-depth statistical analysis. A schematic diagram of the flow of data screening was shown in Figure 1. All procedures were implemented using R software (version 4.3.1).

### 2.3 Statistical analysis

In the statistical analysis, we used a combination of frequency and signal intensity analyses to explore the relationship between baclofen and adverse effects. Disproportionality analysis is an important tool in pharmacovigilance analysis to identify potential safety signals by analyzing the reported frequency of drug-related adverse events compared with the event frequency of other drugs in the overall database (Ding et al., 2025). In this study, multiple disproportionality analysis methods, including frequency method and Bayesian method, were used to comprehensively mine the signals of drug-related ADEs. Signal strength analysis was performed using the following method: Reporting Odds Ratio (ROR): The strength of association between baclofen and adverse effects was calculated. The ROR is a widely utilized statistical method for conducting disequilibrium analysis. It is derived from the principles of a 2 × 2 contingency table (Table 1). The criterion was that the lower limit of the 95% confidence interval of the ROR value was >1. Proportional Reporting Ratio (PRR): A measure of the relative risk of baclofen based on the proportion of adverse reactions reported in the database as a whole. The screening criteria were PRR ≥ 2 and χ^2^ value ≥ 4. Bayesian methods: including Bayesian Confidence Propagation Neural Network (BCPNN) and Multi-Item Gamma Poisson Shrinker (MGPS) were used to evaluate the association strength of rare events (Chen et al., 2024; Shu et al., 2024). Detailed calculation methods are provided in Table 2. ROR can effectively minimize the bias introduced by low-frequency reported events, whereas PRR, owing to its higher specificity, demonstrates superior performance in signal detection during the screening process (Scosyrev et al., 2025). On the other hand, when data is scarce or has missing values, BCPNN can still perform effective signal detection, and as the number of reports increases, its results will tend to stabilize. While MGPS possesses a distinctive advantage in recognizing rare adverse reaction signals. This study integrated the four aforementioned methods, thereby broadening the scope of signal detection and validation. Simultaneously, it effectively minimized false positives via cross-validation, ultimately enhancing the capability to detect rare adverse reactions (Li et al., 2025; Zhu et al., 2025).

**TABLE 1:** A 2 × 2 contingency table for the disproportionality analysis method.

	Baclofen-related ADEs	Non-baclofen-related ADEs	Total
Baclofen	a	b	a + b
Non-baclofen	c	d	c + d
Total	a + c	b + d	N = a + b + c + d

**TABLE 2:** Overview of the ROR, PRR, BCPNN, and EBGM methods, including their formulas and threshold values.

Method	Formula	Threshold
ROR	$\text{ROR}=\frac{a/c}{b/d}$	a≥3
	$\text{SE}\left(\text{lnROR}\right)=\sqrt{\frac{1}{a}+\frac{1}{b}+\frac{1}{c}+\frac{1}{d}}$	ROR ≥3
	$95\%\text{CI}=e^{\ln\left(\text{ROR}\right)\pm 1.96\text{se}}$	95%CI (lower limit) > 1
PRR	$\text{PRR}=\frac{a/\left(a+b\right)}{c/c+d}$	a≥3
	$\text{SE}\left(\text{lnPRR}\right)=\frac{1}{a}-\frac{1}{a+b}+\frac{1}{c}-\frac{1}{c+d}$	ROR ≥2
	$95\%\text{CI}=e^{\ln\left(\text{PRR}\right)\pm 1.96\text{se}}$	95% CI (lower limit) > 1
BCPNN	$\text{IC}=\log_{2}\frac{p\left(x,y\right)}{p\left(x\right)p\left(\gamma \right)}=\log_{2}\frac{a\left(a+b+c+d\right)}{\left(a+b\right)\left(a+c\right)}$	IC025 > 0
	$E\left(\text{IC}\right)=\log_{2}\frac{\left(a+\gamma^{11}\right)\left(a+b+c+d+a\right)\left(a+b+c+d+\beta \right)}{\left(a+b+c+d+\gamma \right)\left(a+b+\alpha 1\right)\left(a+c+\beta 1\right)}$	
	$V\left(\text{IC}\right)=\frac{1}{{\left(\ln2\right)}^{2}}[\frac{\left(a+b+c+d\right)-a+\gamma -\gamma^{11}}{\left(a+\gamma^{11}\right)}+\frac{\left(a+b+c+d\right)-\left(a+b\right)-\alpha 1}{\left(a+b+\alpha 1\right)\left(a+b+c+d+\alpha \right)}+\frac{\left(a+b+c+d+a\right)-\left(a+c\right)+\beta -\beta 1}{\left(a+b+\beta 1\right)\left(1+a+b+c+d+\beta \right)}$	
	$\gamma =\gamma^{11}\frac{\left(a+b+c+d+a\right)\left(a+b+c+d+\beta \right)}{\left(a+b+\alpha 1\right)\left(a+c+\beta 1\right)}$	
	$\text{IC}-2\text{SD}=E\left(\text{IC}\right)-2\sqrt{V\left(\text{IC}\right)}$	
EBGM	$\text{EBGM}=\frac{a\left(a+b+c+d\right)}{\left(a+b\right)\left(a+c\right)}$	EBGM05 > 2
	$\text{SE}\left(\ln\text{EBGM}\right)=\sqrt{\frac{1}{a}+\frac{1}{b}+\frac{1}{c}+\frac{1}{d}}$	
	$95\%\text{CI}=e^{\ln\left(\text{EBGM}\right)\pm 1.96\text{se}}$	

Abbreviations: 95% CI, 95% confidence interval; N, the number of reports; χ^2^, chi-squared; IC, information component; IC025, the lower limit of 95% CI of the IC; EBGM, empirical Bayesian geometric mean; EBGM05, the lower limit of 95% CI of EBGM.

### 2.4 Time-to-onset analysis

The TTO of baclofen-related adverse events was defined as the time difference between the date of the adverse event (EVENT_DT in the DEMO file) and the date of drug initiation (START_DT in the THER file). Cases with inaccurate or missing dates, as well as those with an adverse event occurring prior to the initiation of baclofen treatment, were excluded from the analysis. To ensure data integrity, reports containing erroneous date entries adverse events reported before the treatment start date or incomplete date information was also excluded.

## 3 Results

### 3.1 General characteristics

**FIGURE 2:**
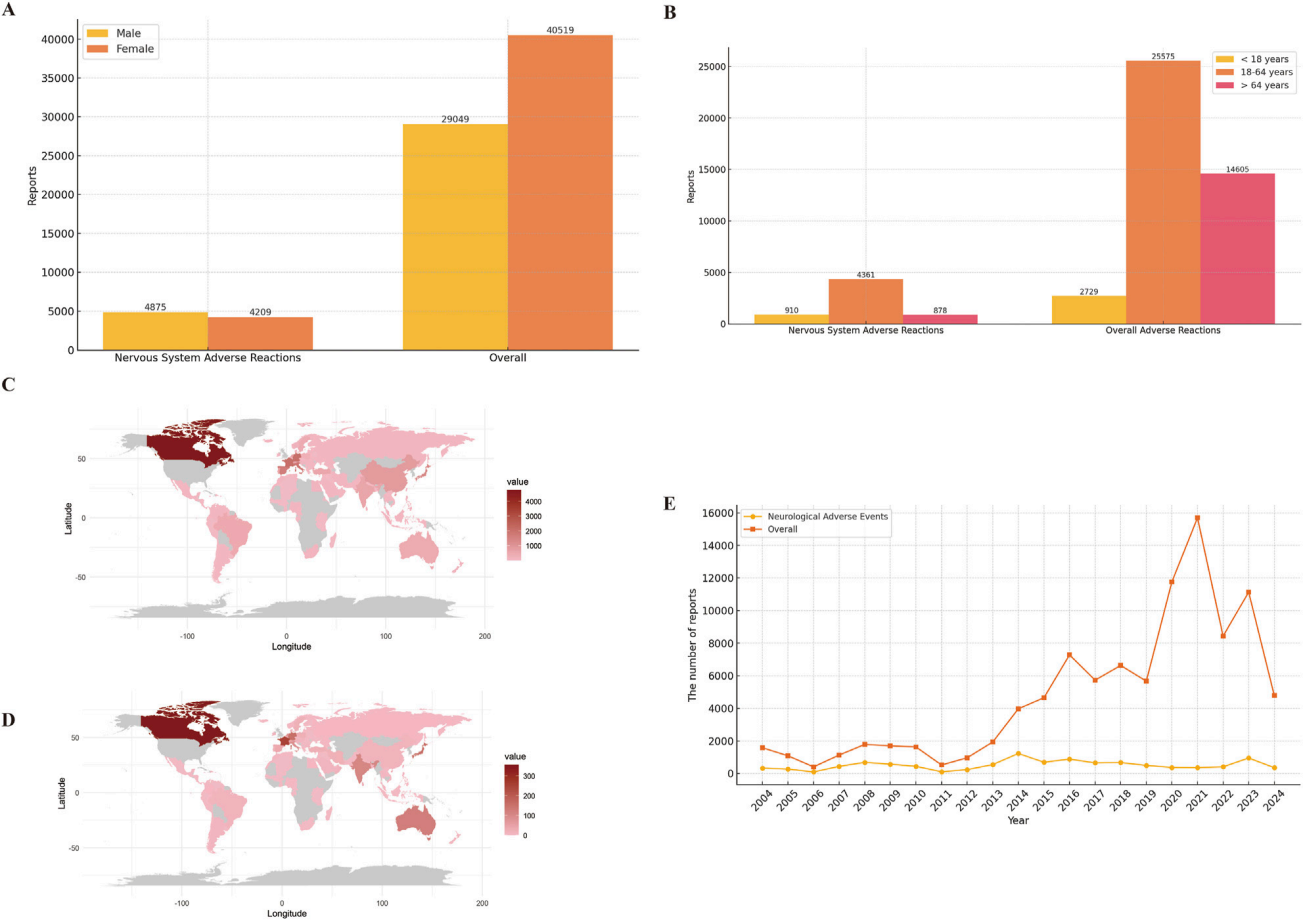
Clinical characteristics of baclofen associated reports from the FAERS database. Footnotes: **(A)** Gender; **(B)** Age; **(C)** Overall adverse drug reactions were reported by country; **(D)** Neurological adverse reactions were reported by country; **(E)** Year distribution of baclofen overall and nervous system adverse reactions reported.

A total of 19,541,994 adverse event reports were available from the FAERS database for the period from the first quarter of 2004 through the third quarter of 2024, after removing duplications. Among these, there were 98,414 baclofen-related adverse events, with 10,685 specifically associated with neurologic adverse events. For all adverse event reports, the gender distribution was as follows: 29,049 (29.5%) were submitted by men and 40,519 (41.2%) by women. Among the 9,084 reports of adverse neurological events, 4,875 (45.6%) involved males, and 4,209 (39.4%) involved females (Figure 2A). The total number of ADEs containing age-specific information was 45,293. Among these, 2,729 (2.8%) were individuals younger than 18 years old, 25,575 (26.0%) were between 18 and 64 years old, and 16,989 (27.2%) were older than 64 years old. For nervous system adverse reactions with age-specific information, the total count was 6,203. Specifically, 910 (8.5%) were younger than 18 years old, 4,361 (40.8%) were between 18 and 64 years old, and 878 (8.7%) were older than 64 years old (Figure 2B). Among the overall adverse events recorded, the countries reporting the highest number of ADEs were the United States (61.9%), followed by the United Kingdom (8.6%), Canada (4.8%), France (2.1%), and Germany (2.1%) (Figure 2C). A similar distribution was observed for the reporting of nervous system adverse events (Figure 2D). The total number of adverse reaction reports for baclofen has exhibited an upward trend, with a peak of 15,688 cases reported in 2021. The number of reported adverse events related to the nervous system associated with baclofen has presented an overall stable trend (Figure 2E). Detailed information is provided in Table 3.

**TABLE 3:** Clinical features of patients reporting baclofen-related neurological adverse events.

Characteristics	Baclofen induced neurological AEs (n = 10,685)		Baclofen induced overall AEs (n = 98,414)	
	Available number	Value	Available number	Value
Gender, n (%)	9,084 (85.0%)		69,568 (70.7%)	
Female		4,209 (39.4%)		40,519 (41.2%)
Male		4,875 (45.6%)		29,049 (29.5%)
Missing		1,601 (15.0%)		28,846 (29.3%)
Age (years), n (%)	6,203 (58.1%)		45,293 (46.0%)	
<18		910 (8.5%)		2,729 (2.8%)
18≤ and <65		4,361 (40.8%)		25,575 (26.0%)
65≤ and <85		878 (8.2%)		14,605 (14.8%)
<85		54 (0.5%)		2,384 (2.4%)
Missing		4,482 (41.9%)		53,121 (54.0%)
Weight (kg), n (%)	1,045 (9.8%)		8,403 (8.5%)	
<50		255 (2.4%)		1,026 (1.0%)
50≤ and ≤100		672 (6.3%)		6,395 (6.5%)
>100		118 (1.1%)		982 (1.0%)
Missing		9,640 (90.2%)		90,011 (91.5%)
Reported countries, n (%)	10,672 (99.9%)		98,344 (99.9%)	
United States		7,758 (72.6%)		60,967 (61.9%)
United Kingdom		181 (1.7%)		8,451 (8.6%)
Canada		356 (3.3%)		4,749 (4.8%)
France		249 (2.3%)		2058 (2.1%)
Germany		153 (1.4%)		2025 (2.1%)
Missing		13 (0.1%)		70 (0.1%)
Outcomes, n (%)	9,416 (87.6%)		50,044 (48.2%)	
Death		443 (4.3%)		4,495 (4.6%)
Life-threatening		753 (7.4%)		3,025 (3.1%)
Hospitalization		3,308 (32.3%)		15,398 (15.6%)
Disability		58 (0.6%)		847 (0.9%)
Other outcomes		3,585 (30.6%)		26,279 (26.7%)
Missing		1,269 (12.4%)		49,952 (51.8%)
Reporters, n (%)	9,567 (89.5%)		94,084 (95.6%)	
Health professional		1,132 (10.6%)		7,697 (7.8%)
Consumer		2,181 (20.4%)		56,504 (57.4%)
Other		6,254 (48.0%)		29,883 (26.0%)
Missing		1,118 (10.5%)		4,330 (4.4%)
Indication	9,229 (85.8%)		90,495 (91.8%)	
Muscle spasticity		4,986 (48.7%)		12,408 (12.9%)
Arthritis		23 (0.2%)		8,054 (8.4%)
Pain		285 (2.8%)		5,946 (6.2%)
Arthralgia		28 (0.3%)		4,300 (4.5%)
Back pain		171 (1.7%)		3,486 (3.6%)
Missing		1,456 (14.2%)		7,919 (8.2%)

### 3.2 Signal detection at the PT level

**FIGURE 3:**
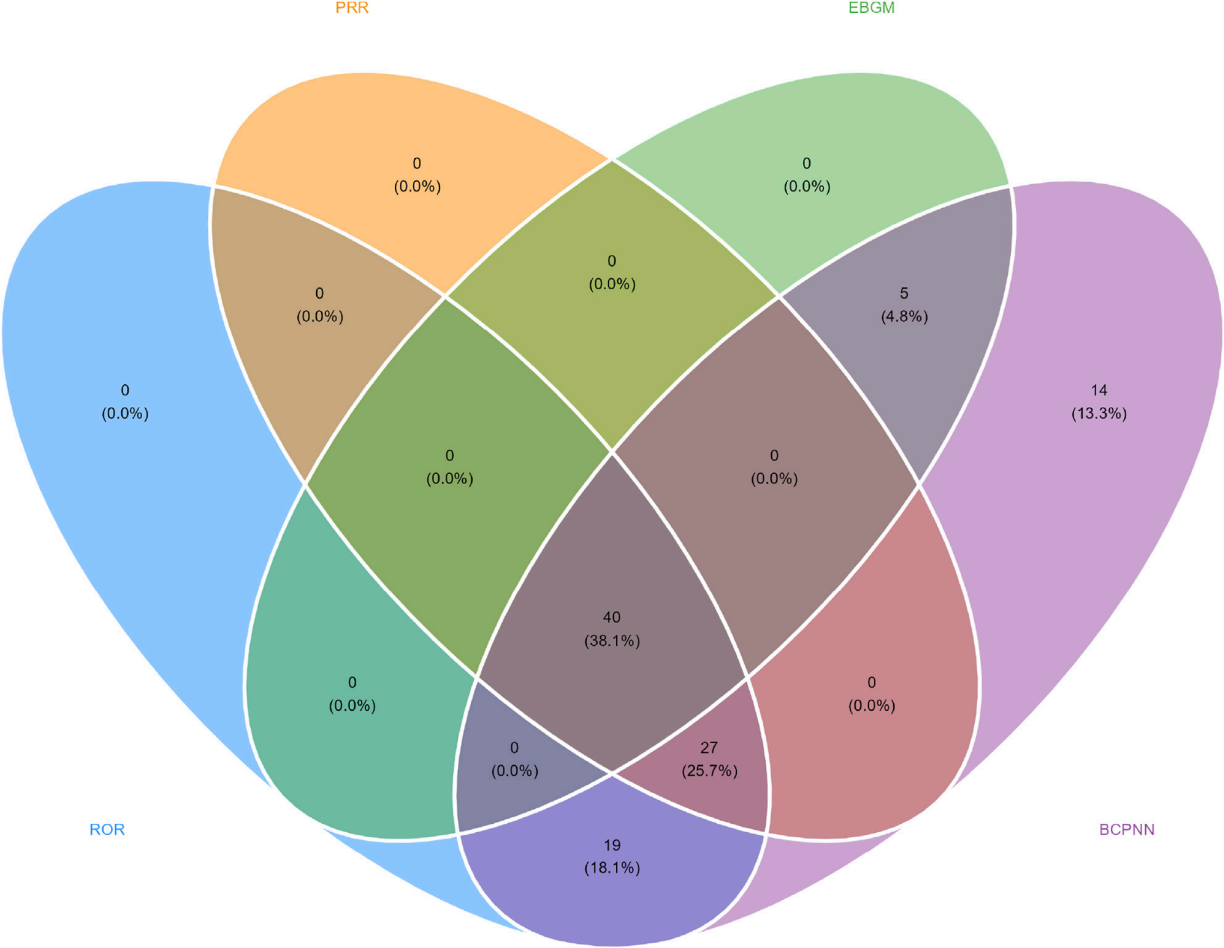
Venn diagram representation of baclofen-Induced nervous system adverse reaction signal values.

**FIGURE 4:**
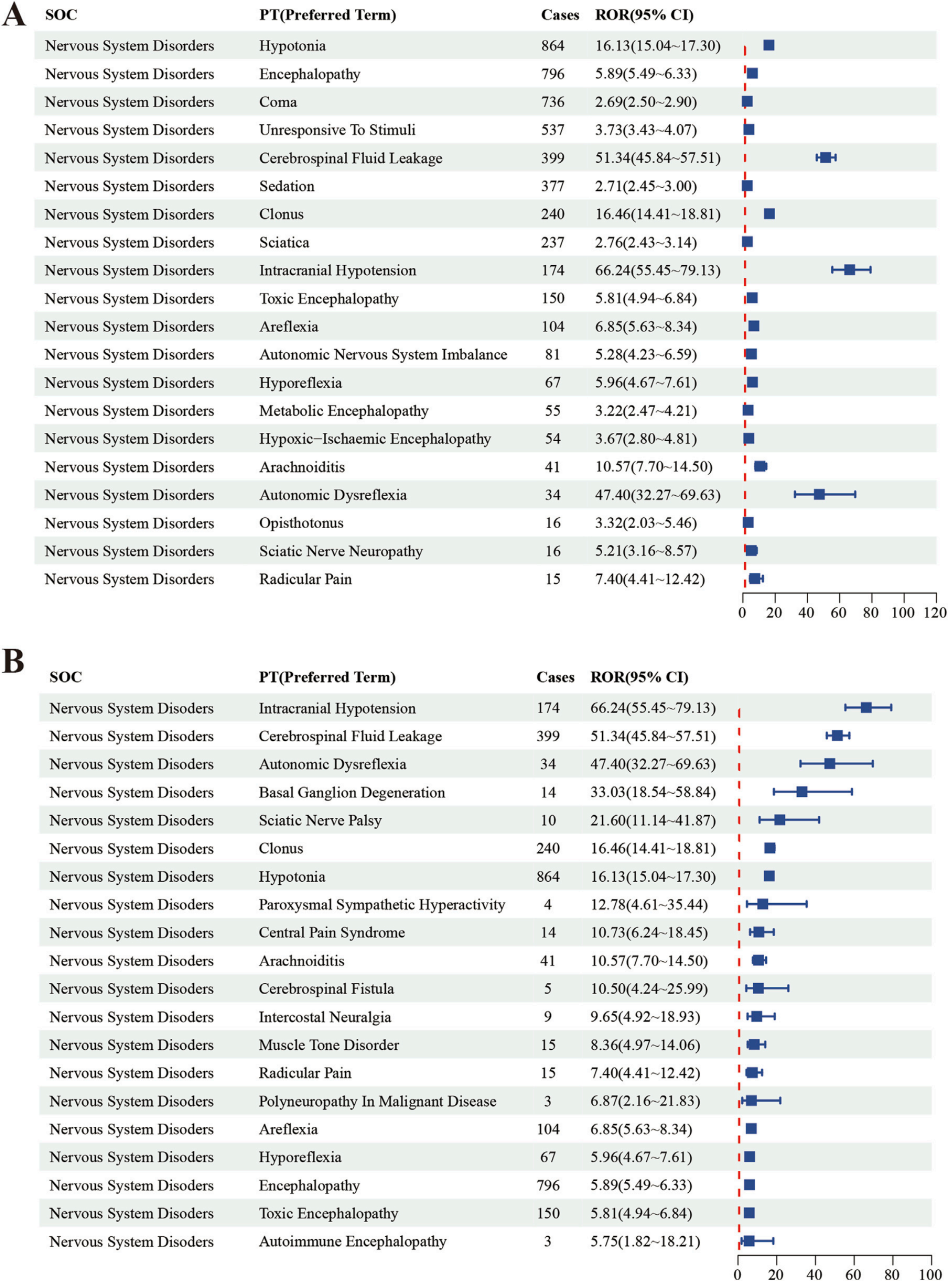
The top 20 PT results for baclofen positivity in ROR. Footnotes: **(A)** The highest signal frequency; **(B)** The highest signal intensity.

In this study, we systematically screened a total of 432 neurologically relevant PTs. To minimize the false positive rates, four signal detection methods were employed, and the pertinent PT was included in the analysis solely when all methods yielded positive results. Ultimately, according to this screening criterion, we identified 40 neurologically relevant PTs associated with baclofen. The Venn diagram in Figure 3 visually illustrated the PTs that met the positive threshold of all four algorithms at the PT level. Table 4 presents the top 20 ADEs associated with baclofen at the PT levels. To enhance the clarity of the data presentation, we visualized the PT signals using a forest plot. The top 5 PTs in terms of the number of adverse events reported were Hypotonia (n = 864), Encephalopathy (n = 796), Coma (n = 736), Unresponsive to stimuli (n = 537), Cerebrospinal fluid leakage (n = 399) (Figure 4A). The top 5 PTs for ROR values are Intracranial hypotension [ROR 66.24 (55.45–79.13)], Cerebrospinal fluid leakage [ROR 51.34 (45.84–57.51)], Autonomic dysreflexia [ROR 47.4 (32.27–69.63)],Basal ganglion degeneration [ROR 33.03 (18.54–58.84)], Sciatic nerve palsy [ROR 21.6 (11.14–41.87)] (Figure 4B).

**TABLE 4:** Signal strength of neurological adverse drug events induced by baclofen at the PT level in the FAERS database.

PT	Case reports	ROR (95% CI)	PRR (95% CI)	IC (IC025)	EBGM (EBGM05)
Hypotonia	864	16.13 (15.04–17.3)	16.09 (11,070.85)	3.87 (3.77)	14.66 (13.82)
Encephalopathy	796	5.89 (5.49–6.33)	5.88 (3,108.73)	2.51 (2.41)	5.7 (5.37)
Coma	736	2.69 (2.5–2.9)	2.69 (768.48)	1.41 (1.3)	2.66 (2.5)
Unresponsive to stimuli	537	3.73 (3.43–4.07)	3.73 (1,047.11)	1.87 (1.75)	3.66 (3.41)
Cerebrospinal fluid leakage	399	51.34 (45.84–57.51)	51.28 (14,742.17)	5.27 (5.11)	38.68 (35.18)
Sedation	377	2.71 (2.45–3)	2.71 (399.26)	1.42 (1.27)	2.68 (2.46)
Clonus	240	16.46 (14.41–18.81)	16.45 (3,145.54)	3.9 (3.71)	14.95 (13.38)
Sciatica	237	2.76 (2.43–3.14)	2.76 (261.4)	1.45 (1.26)	2.73 (2.45)
Intracranial hypotension	174	66.24 (55.45–79.13)	66.21 (7,804.72)	5.54 (5.29)	46.54 (40.11)
Toxic encephalopathy	150	5.81 (4.94–6.84)	5.81 (575.72)	2.49 (2.26)	5.64 (4.92)
Areflexia	104	6.85 (5.63–8.34)	6.85 (497.4)	2.72 (2.43)	6.6 (5.6)
Autonomic nervous system imbalance	81	5.28 (4.23–6.59)	5.28 (271.9)	2.36 (2.04)	5.14 (4.27)
Hyporeflexia	67	5.96 (4.67–7.61)	5.96 (266.17)	2.53 (2.17)	5.77 (4.71)
Metabolic encephalopathy	55	3.22 (2.47–4.21)	3.22 (82.64)	1.67 (1.28)	3.18 (2.54)
Hypoxic-ischaemic encephalopathy	54	3.67 (2.8–4.81)	3.67 (102.62)	1.85 (1.46)	3.61 (2.88)
Arachnoiditis	41	10.57 (7.7–14.5)	10.57 (332.21)	3.31 (2.85)	9.95 (7.63)
Autonomic dysreflexia	34	47.4 (32.27–69.63)	47.4 (1,179.57)	5.19 (4.64)	36.44 (26.41)
Opisthotonus	16	3.32 (2.03–5.46)	3.32 (25.45)	1.71 (1)	3.28 (2.16)
Sciatic nerve neuropathy	16	5.21 (3.16–8.57)	5.21 (52.63)	2.34 (1.63)	5.07 (3.34)
Radicular pain	15	7.4 (4.41–12.42)	7.4 (79.15)	2.83 (2.09)	7.1 (4.6)

PT, preferred terms.

**TABLE 5:** Comparative analysis of clinical characteristics between serious and non-serious adverse event reports.

	Serious cases	Non-serious cases	Statistic	*P*-value
Gender, n			315.57	<0.01
Female	2,110	2099		
Male	2,388	2,487		
Missing	408	1,193		
Types of AEs, n				
Coma	662	74	615.19	<0.01
Unresponsive to stimuli	441	96	296.96	<0.01
Hypotonia	432	432	6.13	<0.05
Depressed level of consciousness	358	108	186.15	<0.01
Lethargy	320	207	48.31	<0.01
Encephalopathy	268	528	51.41	<0.01
Sedation	168	209	0.23	0.63
Sciatica	156	81	37.86	<0.01
Cerebrospinal fluid leakage	146	253	14.12	<0.01
Neurotoxicity	140	66	40.21	<0.01
Altered state of consciousness	128	66	31.21	<0.01
Clonus	127	113	4.56	<0.05
Dystonia	116	96	6.39	<0.05
Toxic encephalopathy	109	41	42.75	<0.01
Status epilepticus	105	12	89.73	<0.01
Generalized tonic-clonic seizure	95	17	67.41	<0.01
Areflexia	92	12	74.83	<0.01
Myoclonus	92	51	19.06	<0.01
Autonomic nervous system imbalance	64	17	34.67	<0.01
Intracranial hypotension	64	110	5.57	<0.05
Hyporeflexia	50	17	21.23	<0.01
Hypoxic-ischaemic encephalopathy	46	8	32.13	<0.01
Metabolic encephalopathy	36	19	7.72	<0.01
Hyperreflexia	28	21	2.06	0.15
Stupor	26	7	13.10	<0.01
Autonomic dysreflexia	23	11	5.63	<0.05
Neurological decompensation	23	3	17.32	<0.01
Quadriplegia	18	3	11.86	<0.01
Orthostatic intolerance	15	0	15.58	<0.01
Arachnoiditis	14	27	1.844	0.17
Spinal cord disorder	13	13	0.049	0.83
Opisthotonus	13	3	6.69	<0.01
Brain stem syndrome	13	2	8.46	<0.01
Sudden onset of sleep	12	10	0.35	0.55
Tonic clonic movements	12	0	12.05	<0.01
Hyporesponsive to stimuli	12	2	7.40	<0.01
Psychogenic seizure	12	2	7.41	<0.01
Basal ganglion degeneration	12	2	7.41	<0.01
Reflexes abnormal	9	3	3.00	0.08
Frontotemporal dementia	9	2	4.36	<0.05
Oromandibular dystonia	8	3	2.20	0.14
Anaesthesia	8	2	3.41	0.07
Tremor neonatal	7	0	6.22	<0.05
Radicular pain	7	8	0	1
Intercostal neuralgia	6	3	0.83	0.36
Neurodegenerative disorder	6	1	3.01	0.08
Intensive care unit acquired weakness	6	0	5.06	<0.05
Locked-in syndrome	6	0	5.06	<0.05
Central pain pyndrome	5	9	0.25	0.62
Hyponatraemic encephalopathy	5	1	2.05	0.15
Athetosis	4	1	1.17	0.28
Myotonia	4	1	1.17	0.28
Postictal Paralysis	4	0	2.79	0.10

AEs, adverse events; n, number of cases.

### 3.3 Analysis of gender-specific differences in baclofen risk signals

**FIGURE 5:**
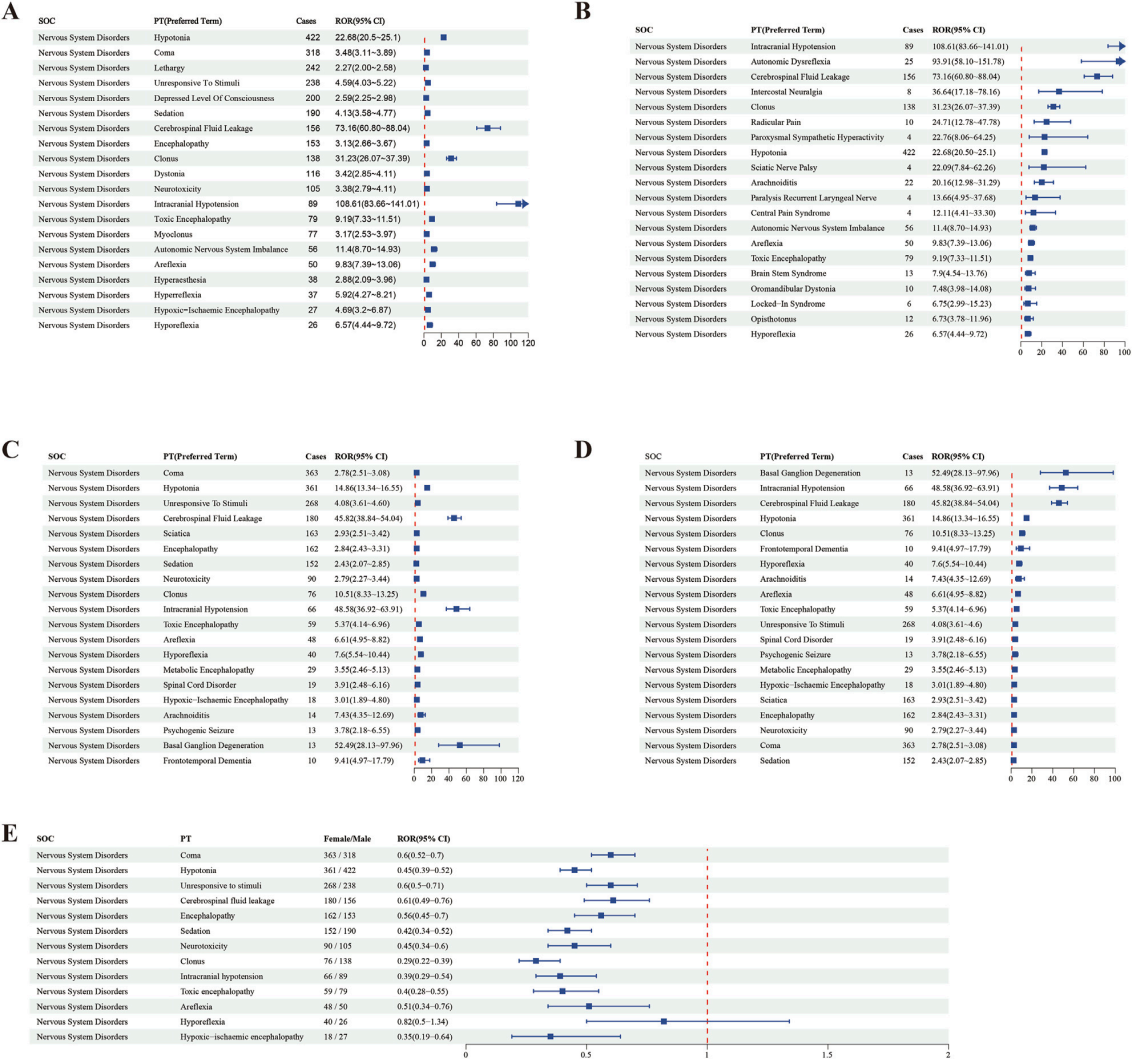
The distribution of ROR signal values and PT factors for baclofen-Induced nervous system adverse reactions by gender. Footnotes: **(A)** The highest signal frequency in males; **(B)** The highest signal intensity in males; **(C)** The highest signal frequency in females; **(D)** The highest signal intensity in females; **(E)** Relative distribution of PT by gender for baclofen-induced nervous system adverse reactions.

To investigate the potential influence of gender on baclofen-induced adverse drug reactions in neurological disorders, we performed an in-depth analysis of various neurology-related ADEs reported in female and male patients. In male patients, the top five most prevalent PTs linked with baclofen use were respectively Hypotonia (n = 422), Coma (n = 318), Lethargy (n = 242), Unresponsive to stimuli (n = 238), Depressed level of consciousness (n = 200) (Figure 5A). The top five adverse reactions in women were Coma (n = 363), Hypotonia (n = 361), Unresponsive to stimuli (n = 268), Cerebrospinal fluid leakage (n = 180), Sciatica (n = 163) (Figure 5C). In male patients, the top five major factors related to baclofen that met all four calculation methods were Low Intracranial pressure [ROR 108.61 (83.66–141.01)], Abnormal autonomic reflex [ROR 93.91 (58.1–151.78)], Cerebrospinal fluid leakage [ROR 73.16 (60.8–88.04)], Intercostal neuralgia [ROR 36.64 (17.18–78.16)], and Clonus [ROR 31.23 (26.07–37.39)] (Figure 5B). In female patients, the top five major PT signals of the nervous system associated with baclofen that met all four calculation methods were Basal ganglion degeneration [52.49 (28.13–97.96)], Low intracranial pressure [ROR 48.58 (36.92–63.91)], Cerebrospinal fluid leakage [ROR 45.82 (38.84–54.04)], Sciatic nerve paralysis [ROR 22.83 (8.03–64.95)], and Abnormal autonomic reflex [ROR 18.81 (7.46–47.44)] (Figure 5D).

This study provides a detailed analysis of the proportions of male and female patients experiencing baclofen-related nervous system adverse reactions, along with their gender-based differences. The names of each PT, the corresponding female-to-male patient-reported number ratio (female/male), the ROR, and the 95% confidence interval are presented in forest plots (Figure 5E). Blue squares represent the ROR values for each adverse reaction, horizontal lines denote the 95% confidence intervals, and a red dashed line serves as the baseline for ROR = 1, indicating an equal reporting ratio between genders. The results indicate that the confidence intervals for many adverse reactions lie entirely to the left of ROR = 1, suggesting a significantly lower proportion of adverse event reports from women compared to men. For instance, Coma (ROR = 0.6, 95% CI: 0.52–0.7), Hypotonia (ROR = 0.45,95% CI: 0.39–0.52), Unresponsive to Stimuli (ROR = 0.6,95% CI:0.50–0.71), Cerebrospinal fluid leakage (ROR = 0.61,95% CI: 0.49–0.76), Encephalopathy (ROR = 0.56,95% CI: 0.45–0.70), demonstrating that the reporting proportion of females is conspicuously lower than that of males. Additionally, the confidence intervals of some adverse reactions traverse ROR = 1, such as Hyporeflexia (ROR = 0.82, 95% CI: 0.5–1.34), indicating that there is no marked difference in the reporting of these adverse reactions between males and females. Through such data and graphical analyses, the distribution characteristics of adverse reactions reported by male and female patients are lucidly disclosed.

### 3.4 Time-to-onset analysis

After removing reports with inaccuracies, missing information, or unknown details, 434 ADEs were included, with a median TTO of 27 days. The majority of cases occurred within the first 0–30 days of baclofen use (n = 241, 55.5%). There were 35 cases (8.1%) within 30–60 days, 21 cases (4.8%) within 60–90 days, 14 cases (3.2%) within 90–120 days, 7 cases (1.6%) within 120–150 days, 13 cases (3%) within 150–180 days, and 20 cases (4.6%) within 181–360 days. Notably, ADEs were still observed in 83 cases (19.1%) after 1 year of baclofen treatment.

### 3.5 Serious vs. non-serious cases: baclofen neurological adverse events

There was a statistically significant difference in the gender ratio between severe and non-severe cases (p < 0.01). Among severe cases, 2,388 were male (48.7%), which was higher than non-severe cases (n = 2,487, 43.1%). In the same way, the proportion of females was slightly higher in severe cases (n = 2,110, 44.1%) compared to non-severe cases (n = 2,099, 43.1%). In severe cases, the top five neurological adverse reactions identified consistently by all four signal detection methods were: Coma (n = 662), Unresponsive to stimuli (n = 441), Hypotonia (n = 432), Depressed level of consciousness (n = 358), and Lethargy (n = 320). Among non-severe adverse reactions, the top five neurological adverse reactions confirmed by all four signal detection methods included Encephalopathy (n = 528), Hypotonia (n = 432), Cerebrospinal fluid leakage (n = 253), Somnolence (n = 225), and Sedation (n = 209). Differences in clinical characteristics of serious and non-serious reports were shown in (Table 5).

## 4 Discussion

To our knowledge, this study is the first to employ the FAERS database to disclose the distribution of neurological adverse reactions associated with baclofen, offering a significant reference for pharmacovigilance and clinical risk management. In this research, we placed emphasis on the clinical characteristics of baclofen in the nervous system, gender disparities, TTO, and the distinctions between severe and non-severe cases. The occurrence of adverse reactions in the nervous system can have a significant impact on patients’ health, underscoring the critical need for healthcare professionals to increase their awareness and improve related diagnostic and management skills.

The results of our analysis demonstrated a significant upward trend in baclofen-related ADEs, with the total number of reported incidents peaking at 15,688 in 2021, accounting for 15.9% of all reported ADEs. This growth trend may be closely associated with the expansion of the approved indication range for baclofen and its widespread adoption in clinical practice (Creamer et al., 2018a; Bonouvrié et al., 2019; Romito et al., 2021). However, it is notable that although the overall quantity of ADEs has increased, the number of ADEs associated with neurological adverse reactions has not presented a significant changing trend. This phenomenon could be ascribed to the elevated awareness of medication safety among patients and the continuous refinement of the clinical medication monitoring system. Although no significant increase in ADEs related to the nervous system has been observed with baclofen, it can still cause a variety of serious neurological adverse reactions, including coma, and may even lead to death. Therefore, in clinical practice, high attention should still be paid to the adverse neurological reactions of baclofen.

Our real-world data reveal that among the neurological adverse reactions, the top five most frequently reported PTs meeting all four algorithms were Hypotonia (n = 864), Encephalopathy (n = 796), Coma (n = 736), Unresponsive to stimuli (n = 537), Cerebrospinal fluid leakage (n = 399). This is in accordance with previous research findings. For example, in a real-world cohort study, the use of baclofen was associated with a higher risk of encephalopathy compared with the use of tizanidine or cyclobenzaprine (Hwang et al., 2023). The kidney plays a crucial role in baclofen elimination, accounting for approximately 70% of its clearance (Miller, 2017). Consequently, renal impairment may result in elevated serum concentrations of baclofen (Meillier et al., 2015). Physicians should take into account the potential toxicity of baclofen in patients with impaired renal function and altered mental status following its administration. For instance, a case report described a 57-year-old patient with end-stage renal disease who experienced progressive confusion and generalized hypotonia following the administration of baclofen (Marquez, 2015). Furthermore, in a population-based cohort study involving 360 elderly patients undergoing maintenance dialysis, 24 patients developed encephalopathy as a complication following baclofen administration and subsequently required hospitalization (Chauvin et al., 2020). Baclofen is a muscle relaxant that acts primarily in the spinal cord through the activation of inhibitory GABA-B receptors. Due to the moderate lipophilicity of baclofen, its ability to penetrate the blood-brain barrier is somewhat limited, so a higher oral dose is required to achieve the desired therapeutic effect (Romito et al., 2021). However, excessive intake of baclofen may lead to adverse reactions in the nervous system, such as drowsiness and even coma. In a retrospective analysis of 37 patients, the common clinical manifestations of baclofen poisoning included encephalopathy, coma as well as respiratory depression, hyporeflexia, and autonomic dysfunction (Creamer et al., 2018b). This is in line with our research findings. Furthermore, our analysis unexpectedly revealed several central nervous system adverse reactions, including Cerebrospinal fluid leakage [n = 399, ROR 51.34 (45.84–57.51)] and Intracranial hypotension [n = 174, ROR 66.24 (55.45–79.13)]. Some studies have shown that intrathecal baclofen (ITB) can lead to Cerebrospinal fluid leakage (Bonouvrié et al., 2022). In a 7-year retrospective study, among all patients who received intrathecal baclofen therapy, 21% developed cerebrospinal fluid leakage (Imerci et al., 2020b). When cerebrospinal fluid leakage occurs, patients have more perioperative adverse events and a higher 90-day readmission rate compared with those without these complication (Imerci et al., 2020b). Excessive cerebrospinal fluid leakage may also lead to intracranial hypotension (Wilson et al., 2023). A blood patch can be performed to prevent Cerebrospinal fluid leakage, and indeed, some surgical teams do this systematically (Imerci et al., 2020a). Epidural blood patches (EBPs) are also frequently performed in children with cerebral palsy (CP) to manage post-dural puncture headache (PDPH) due to cerebrospinal fluid leaks after ITB placement (Imerci et al., 2019). Additionally, we observed an increased number of reports for conditions such as Encephalopathy, Toxic encephalopathy, Metabolic encephalopathy, Hypoxic-ischemic encephalopathy, and Arachnoiditis. We identified several previously underreported adverse events, including Sciatica (n = 32), Hyporeflexia (n = 67), Autonomic nervous system dysfunction (n = 81), Opisthotonus (n = 16), and Radicular pain (n = 16). These findings were infrequently documented in both animal experiments and clinical trials (Karol et al., 2011).

Analysis of gender differences reveals that among all ADEs associated with baclofen, the proportion of female patients (41.20%) is significantly higher than that of male patients (29.50%). This discrepancy could potentially be associated with alcohol use disorder, as well as the primary indications for baclofen, including muscle spasms and neuralgia. As a GABA-B receptor agonist, baclofen has been proposed as a potential therapeutic option for the treatment of alcohol use disorders (Garbutt et al., 2021; Agabio et al., 2023). The significant increase in female alcohol dependence cases in recent years may be one of the important reasons for the gender difference in the reporting of adverse effects of baclofen (Khemiri et al., 2016). In addition, the main indications for baclofen, such as muscle spasms (Limerick et al., 2023) and multiple sclerosis (McGinley et al., 2021), are generally more common in women, which may also be another contributing factor. Our study further indicates that, in comparison with females, males constitute a marginally higher proportion of the neurological adverse reactions related to baclofen. However, the number of relevant studies is limited, and additional research is warranted to elucidate the specific mechanisms underlying these gender differences. In addition, the FAERS data may be susceptible to reporting bias, particularly in the context of sex-specific data. Since FAERS data collection is based on voluntary reporting by healthcare professionals and the general public, disparities in reporting frequency between genders could exist, which might influence the accuracy and comparability of the data (Guan et al., 2024).

According to the statistical analysis, the TTO of baclofen-induced neurological adverse reactions exhibited a significant temporal clustering. The majority of these adverse effects were observed in the early stages of treatment, particularly within the first 30 days. Furthermore, the TTO distribution for specific adverse reactions demonstrated notable variations (Heetla et al., 2014). For rapidly induced reactions, such as encephalopathy and Coma, approximately 75% of cases occurred within the first 2 weeks of medication. Rapidly induced adverse reactions may be related to the current administration methods of baclofen pumps or intrathecal injections (Shlobin et al., 2023; Cespedes et al., 2024; AlShaqaq et al., 2025). For baclofen pumps, mechanical drug delivery systems provide distinct therapeutic advantages; however, they are also susceptible to human error or equipment malfunctions. Both scenarios can result in drug overdose, insufficient dosage, or abrupt drug withdrawal, which may lead to severe health consequences (Caron et al., 2014; Romito et al., 2021; Fu and Perloff, 2022). Muscle spasticity, on the other hand, is a delayed adverse reaction with a significantly prolonged median induction time of 151 days. The data suggests that such reactions may be related to long-term medication and require continuous monitoring throughout the treatment period. Additionally, a few cases have shown significant long-term delayed effects; for instance, case reports indicate that some neurological adverse reactions may still occur even after 1 year of medication.

Our study found that baclofen use was associated with a higher number of serious adverse events, with a significantly higher proportion of serious adverse events than nonserious adverse events in both men and women. In a cohort study involving approximately 47,000 patients treated with baclofen for alcohol use disorder (AUD), which utilized a French health insurance claims database, the use of baclofen was associated with a significantly higher risk of hospitalization and mortality compared to other approved medications (Chaignot et al., 2018). In addition, a study conducted in Australia examining 102 baclofen-related fatalities revealed that the mean age at death was below 50 years and that intentional poisoning constituted the most prevalent cause of death (Zahra et al., 2024). Multiple studies have shown that people with alcohol use disorders are at higher risk when using baclofen, especially those individuals with a history of self-harm, suicide attempts, or substance use disorder (Holla et al., 2015; Beraha et al., 2016; Auffret et al., 2017). Therefore, healthcare providers should pay special attention to these high-risk groups and conduct a comprehensive assessment before starting baclofen treatment. Baclofen withdrawal syndrome is one of the most concerned complications during baclofen treatment, which can progress rapidly and has a high morbidity and mortality. Abrupt and drastic discontinuation or reduction of medication may trigger withdrawal symptoms, including changes in mental state, increased convulsions, fever, nausea, fatigue, etc. In severe cases, it may even lead to coma (Lad et al., 2008; Caron et al., 2014). Baclofen causes slow synaptic depression by activating G protein-coupled GABAB receptors, increasing potassium conductance in neuronal membranes, and reducing calcium influx at presynaptic terminal (Marquez, 2015). This mechanism results in a slowing of the hyperpolarization of neurons and a reduction in the release of excitatory neurotransmitters, which in turn reduces the number and amplitude of excitatory postsynaptic potentials along the dendrites of motor neurons (Salim et al., 2018). However, abrupt withdrawal of the drug may trigger rebound excitation at all levels of the neural axis, leading to a series of serious side effects (Pierce et al., 2018). For baclofen withdrawal syndrome, emergency resumption of medication should be carried out promptly, and the route of administration and dosage should remain the same as before the interruption (Romito et al., 2021).

This study conducted a systematic analysis of neurological adverse reactions of baclofen based on the FAERS database. First, we identified potential neurologic adverse events that may have been triggered by baclofen. Second, adverse events with significant signals were explored in depth. This study not only provides valuable insights into the neurological adverse effects of baclofen but also provides a new theoretical basis for evaluating the safety of this widely used class of drugs. In addition, this study points the way for future pharmacovigilance analyses and clinical trials that can help to further validate our findings and elucidate the mechanisms of associated neurological adverse effects.

Nevertheless, this study possesses limitations. The FAERS database bears inherent defects, including under-reporting of adverse events and over-reporting of events unassociated with drugs (Xia et al., 2024). The quality and accuracy of the reports hinge upon the expertise of the reporters Furthermore, the majority of the data results stem from countries such as the United States and those in the West, and the results might not be applicable to Asian regions like China (Li R. et al., 2024). Ultimately, the restricted and regionally concentrated data demand more extensive and long-term studies to validate these discoveries.

## 5 Conclusion

In conclusion, this study performed an in-depth analysis of baclofen-associated neurological adverse events using real-world data from the FAERS database. Moreover, the major adverse effects identified in this study align with those listed on the baclofen product label; however, several potential central nervous system adverse effects, such as Cerebrospinal fluid leakage and Intracranial hypotension, which have not been widely recognized, were also detected. Our results enhance the understanding of baclofen-related neurotoxicity and provide valuable insights for healthcare professionals, aiding them in reducing the risk of adverse events more effectively through postmarketing safety evaluation.

## Data Availability

The datasets presented in this study can be found in online repositories. The names of the repository/repositories and accession number(s) can be found in the article/Supplementary Material.
